# Indents

**Published:** 1915-06

**Authors:** 


### INDENTS.

---

☞ **Brief Articles of Technical or Scientific Value will receive notice and comment. Contributions invited.**

---

*The Forsythe Infirmary and Saving the Teeth of Boston Children.* After discussing the purposes of the management of the Forsythe Infirmary, Dr. Ottolengui, in the January *Items of Interest,* makes the following sensible comment: Let every school in Boston be supplied with properly trained dental nurses, whose duty it shall be to cleanse the teeth of the children once a month, and to train them in mouth hygiene, at the same time noting and charting incipient caries. The Forsythe Dental Infirmary could then readily treat all the cases of caries as fast as discovered. This, of course, can not be accomplished in a single year, but the plan can be worked out so as to practically eradicate caries and more especially foul mouths, exposed pulps and abscessed teeth within ten years. Would this be worth while?

The plan in detail is as follows: Abandon at once the hopeless undertaking of filling teeth for all the school children of Boston. Last year Boston had no such infirmary; the children who already have decayed teeth will therefore be no worse off now than they were then, if the infirmary should not undertake the filling of their teeth.

Add to the present dental staff of operators fifty or sixty women, who shall constitute the first class of trained dental nurses.

Limit the work of these women during this first year of training to operations upon the infant classes. These youngsters, having few if any permanent teeth, can suffer little harm from these women in training, less harm, surely, than the patients in hospitals attended by under-graduate nurses. Let these dental nurses, after graduation, be given chairs in public school buildings, where they could continue the prophylactic work on the same children, now one grade higher in their school work, and let a new class of nurses be received into the Infirmary Training School. Then year by year more and more nurses should be trained by the Forsythe Infirmary and sent into the school infirmaries, and by the time the first children thus treated are ready to enter high school, they will do so with practically clean mouths; with teeth, when filled at all, filled with small fillings; with all or nearly all teeth having sound, living and healthy pulps; with no abscessed teeth present. With trained prophylactic dental experts, caring for the mouth hygiene of all children to the top of the grammar grade; with all children entering high school thoroughly appreciative of the value of sound teeth and skilled in the care of them; then would the Forsythe Dental Infirmary easily be able to care for the cavities in children's teeth as fast as their own graduate dental nurses find and report them. Then would the problem have been solved.

*New Jersey Societies.* The New Jersey dentists are wide awake with the spirit of the times in the matter of organized society work. There are fourteen societies in existence in that state in spite of Hoboken.

*Applied Science and Dentistry.* The advance of applied science, and particularly of chemistry, physics and bacteriology, within the past seventy years, has nowhere taken effect more advantageously than in dental medicine and the dental art. American inventiveness took effect chiefly on the tools and mechanical processes of dentistry. German chemical science made valuable additions to the materials with which teeth are filled, and the electric current made possible the use of machine drills. Bacteriology has shed a flood of light on the processes of inflammation and suppuration, and on the methods of contagion or infection; and both chemistry and physics have supplied various means of preventing and diminishing pains in dental operations.

These improvements in the science and art of dentistry have enabled the profession to do for individuals much more than they were formerly able to do for the preservation of health and prolongation of life; but simultaneously with this larger possibility of service has gone the greater cost of the service; so that the skillful treatment of the teeth from childhood to age has become more and more the privilege of the well-to-do, the poor being unable to pay for the costly labors of the accomplished dentist. A clear perception of the deprivations which the less fortunate or successful portion of the community suffers in this respect has led to the establishment and endowment of this infirmary for children. In this beautiful and perfectly equipped building, the children of persons whose earnings are not much more than sufficient to cover the ordinary expenses of their families, are to obtain, at merely nominal cost, as skillful dental service as the well-do-do can buy for their children; and through the service of trained dental nurses the persons responsible at home for the children here treated will be taught how to keep the children's mouths in as good order as their general health permits.

In my view, the teaching function of this institution will be the most telling part of its total work. It is well to put a child's teeth in good order for once and at the moment the child leaves the dental chair; but it is better to teach the mother or sister at home how to keep that child's mouth in good order.

The Forsythe Institute is a monument as well as an infirmary and school. It perfectly illustrates one of the admirable traits of successful business men in the United States—the desire on their part to make use of their private earnings and accumulations to advance some benficial public undertaking. It also illustrates fraternal love and concord. Long may it stand to speak to coming generations of these fine human qualities and to relieve pain, promote health and prolong life.—Dr. Charles W. Eliot, in *Journal of Allied Societies.*

*The Application of Radio-Active Substances in Dentistry.* Radium was introduced into dental therapeutics in 1912, by M. Levy of Berlin. Aside from his numerous publications, the writings of Walkhoff, Trauner, Mamlok, Léger-Dorez, Warnekros, and many others are available to the inquiring student. According to Levy, the following oral diseases have been subjected to radium emanation: Psoriasis of the oral mucous membrane, pyrrohea alveolaris, loosening of the teeth without the presence of pus, marginal gingivitis, leukoplakia, chronic aphthæ, fistulas, and ulcerative stomatitis caused by gout. The therapeutic application of radio-active substances about the mouth may be accomplished by utilizing the following methods and means: The drink cure, mouth-washes, tooth-pastes, compresses, injections, irrigation, inhalation, and finally, variable combinations of these procedures. The drink cure and the application of the mouth-washes are probably the two most prominent means of utilizing radium emanation for such purposes; the other enumerated methods are of questionable value. The technique of the various methods is comparatively simple. As a drink cure, Levy recommends the following procedure: Water charged with emanation, or water containing a specific quantity of a soluble radium salt may be used. The radium content should correspond to about a thousand to three thousand Mache units per day, although higher concentrations have been used with no deleterious side-effects. Every twenty to thirty minutes during the two or three hours following the three main meals, a small quantity of the charged water should be taken. The object is to furnish the organism with small quantities of the products of decomposition of radium, which are slowly absorbed. In due time they reach the blood current and finally are eliminated, primarily through the lungs and to a less extent by the urine, the skin, perspiration, and the saliva. Within twenty minutes after partaking of 600 Mache units, radium emanation has been shown to be present in the saliva. As a gargle, Trauner recommends the following procedure: A quart of water containing about 375 Mache units forms the basis of the mouth-wash. Of this solution, the patient uses two glassfuls (about ten fluid ounces each) per day as a mouth-wash, observing the following precautions: Every dose of the solution—which should not be too large, so as to find ample room in the mouth—has to be worked forcibly between the cheeks and the teeth for at least a minute and a half, so as to de-emanize the water. The water should then be removed from the mouth slowly and in a thin stream. The emanation will separate from the water and precipitate itself upon the mucous surfaces of the mouth. From twenty to thirty minutes are necessary to use up the content of a glassful of the solution. After the gargling, the patient should not eat or drink, and if possible should not speak, for at least one or better two hours, to retain the gaseous emanation in the mouth. With this simple procedure Trauner claims to have obtained most remarkable results. The formation of pus and subjective symptoms are checked in two or three days, remaining only, and to a milder degree, upon those places where accumulations of calcarious deposits are present. The tartar has to be removed thoroughly, and at future sittings careful examination has to be made for remnants of tartar, which represent a constant and sure source of pus production. Large-sized pockets are successfully treated by syringing with two cubic centimeters of a concentrated emanation solution.

If one wishes to obtain a comprehensive view of the present state of radium emanation in therapeutics, one is at once confronted with the enormous mass of accumulated literature. Within the last three years, aside from numerous exhaustive treatises, more than a thousand articles have appeared in current professional journals. The early glowing reports of 1912 and 1913, which in many instances bordered on therapeutic hysteria, fortunately have been superseded by the more sane and unbiased observations of broadminded practitioners. Radium treatment is slowly settling down to the specific phase in medical practice to which undoubtedly it is entitled. During the year's sojourn in Europe (1913-14), the writer had the opportunity to familiarize himself with the present status of the application of radio-active substances as employed in the alleviation of dental diseases. The two dental institutes which have primarily investigated radium therapy are the two institutes of the University of Berlin and of Graz. At the Berlin Institute, Zahnarzt Mamlok, and at Graz Professor Trauner, have carried out extensive investigations on the subject, and both gentlemen have been most courteous in furnishing patients and offering their experience to the writer, which he hereby gratefully acknowledges. Parenthetically, it may be remarked that both gentlemen are close observers and sound in their judgment. Trauner is still an ardent advocate of radium therapy, and he is convinced that its influence is very marked in the treatment of inflammatory conditions of the oral mucous membrane. On the other hand, Mamlok is rather skeptical at present, and he sums up his experience by stating that the prolonged utilization of water charged with radium emanation has a tendency to lower the virulence of the ordinary pus bacteria usually found in inflammatory conditions of the mouth. He has obtained good results in the treatment of pyrrohea alveolaris by combining the following procedures: Careful removal of all tartar deposits, establishing perfect occlusion, splinting of loose teeth, application of redio-active substances, and rigid oral hygiene. It was observed that patients who suffered with pain in connection with their dental ailments were unanimous in their statements that washing with radium-charged water relieved this condition, like "magic," as they expressed it. Of the many other benefits claimed by dental practitioners and patients alike relative to the therapeutic effects of radium mouth-washes, pastes, etc., the writer is extremely skeptical. He has not been able to observe any special value derived from such procedures. In a number of counter-tests, in which a warm physiologic salt solution was substituted for the radium preparations, the comparative results obtained were equally as good. At the meeting of the German National Dental Society, the Central Verein, held in May, 1913, the enthusiasm relative to radium therapy probably had reached its zenith. It had leaped up in sky-rocket fashion, and, following the law of gravitation, came down at an equally rapid rate. At the 1914 meeting of the same society, aside from a few derogatory remarks, one noticed a conspicuous absence of discussion on this supposed panacea for all bodily ills. As the writer has stated above, radium seems to be entitled to a legitimate place in general therapeutics. So far as its application in the treatment of oral diseases, especially pyorrhea alveolaris, is concerned—for which it has received the bulk of its indorsement—at present no positive results can be recorded.—H. Prinz, in *Cosmos.*

*Bone Development in cases of Infra-occlusion.*

I am about finishing a very bad case of infra-occlusion, and I was at one time very much disturbed because I thought I might draw the teeth entirely out of their sockets. I went down to consult Dr. Waldron, as I do when I get in trouble, and we talked the matter over. I then applied what I considered the right degree of pressure on the six anterior teeth in either jaw, and that gentle pressure has been on now for nearly a year, and the infra-occlusion has been entirely corrected and the bite is normal in front. I measured those teeth from the cutting edge of the teeth to the cervical margin of the gum, and I found there is not a millimeter of elongation of those teeth from their sockets. Therefore, I must have brought down the process of the upper jaw and brought up the process in the lower jaw, and they have followed the teeth, and the bone has developed and grown. I speak of that because in such cases we sometimes feel there is danger of the teeth being extruded, but if the pressure is not too strong and is applied in the right way, and the case well watched, we need not fear.—Dr. Taylor, in *Items of Interest.*

*Value of Normal Occlusion.* In order to have a normal and healthy growth of the maxillary bones they must be subjected to normal strain in all directions. This, in my opinion, means that we must discard the time-honored flat amalgam filling and substitute gold inlays, properly carved in the wax pattern.

It may be urged that the cost of the inlay is too great and that many patients can not afford to have the best possible work we can supply. It is, I believe, all a matter of education; what we must do to best serve the public is to teach them the prime importance of good dentistry. Once a layman appreciates the importance of proper occlusion he will find a way to afford to pay for it.

Proper occlusion means, more than any other factor, a clean mouth and healthy oral mucous membrane, good mastication, good digestion and proper assimilation.—Dr. Stearns, in *Items of Interest.*

*A Way to Keep Oil of Cajeput.* Many practitioners use oil of cajeput or some other oil for smoothing off the wax model for an inlay before removing from the cavity. To prevent using an excess and spilling the contents of the bottle, take an ordinary medicine bottle, fill with absorbent cotton. Pour oil on this cotton until slightly saturated. When the pellet of cotton, held in the cotton carrier, is immersed into the oily cotton of the bottle it will not become oversaturated.—F. S. Dilger, in *Review.*

*To Sterilize Instruments.* Use an oblong agate affair bought at hardware store, just right size to hold forceps, etc., fill with water, using about a teaspoonful or more of common washing soda costing about one or two cents per pound; remove forceps after boiling and when right to handle polish with towel; will look like new and there will be no "rusting."—C. P. S. in *Review*.

*To Prevent Slipping of Engine Cord.* When your engine pulley becomes worn so that the cord inclines to slip upon it, fill the bottoms of the grooves with the unvulcanized rubber and note benefit.—V. C. Smedley, in *Digest*.

